# Exploring the small-scale spatial distribution of hypertension and its association to area deprivation based on health insurance claims in Northeastern Germany

**DOI:** 10.1186/s12889-017-5017-x

**Published:** 2018-01-10

**Authors:** B. Kauhl, W. Maier, J. Schweikart, A. Keste, M. Moskwyn

**Affiliations:** 1 AOK Nordost – Die Gesundheitskasse, Department of Medical Care, Berlin, Germany; 2 Beuth University of Applied Sciences, Department III, Civil Engineering and Geoinformatics, Berlin, Germany; 3 Helmholtz Zentrum München, German Research Center for Environmental Health (GmbH), Institute of Health Economics and Health Care Management, Neuherberg, Germany

**Keywords:** Hypertension, Healthcare, Germany, Spatial analysis, Geographically weighted regression, German index of multiple deprivation, Health insurance claims, AOK Nordost

## Abstract

**Background:**

Hypertension is one of the most frequently diagnosed chronic conditions in Germany. Targeted prevention strategies and allocation of general practitioners where they are needed most are necessary to prevent severe complications arising from high blood pressure. However, data on chronic diseases in Germany are mostly available through survey data, which do not only underestimate the actual prevalence but are also only available on coarse spatial scales. The discussion of including area deprivation for planning of healthcare is still relatively young in Germany, although previous studies have shown that area deprivation is associated with adverse health outcomes, irrespective of individual characteristics. The aim of this study is therefore to analyze the spatial distribution of hypertension at very fine geographic scales and to assess location-specific associations between hypertension, socio-demographic population characteristics and area deprivation based on health insurance claims of the AOK Nordost.

**Methods:**

To visualize the spatial distribution of hypertension prevalence at very fine geographic scales, we used the conditional autoregressive Besag–York–Mollié (BYM) model. Geographically weighted regression modelling (GWR) was applied to analyze the location-specific association of hypertension to area deprivation and further socio-demographic population characteristics.

**Results:**

The sex- and age-adjusted prevalence of hypertension was 33.1% in 2012 and varied widely across northeastern Germany. The main risk factors for hypertension were proportions of insurants aged 45–64, 65 and older, area deprivation and proportion of persons commuting to work outside their residential municipality. The GWR model revealed important regional variations in the strength of the examined associations.

**Conclusion:**

Area deprivation has only a significant and therefore direct influence in large parts of Mecklenburg-West Pomerania. However, the spatially varying strength of the association between demographic variables and hypertension indicates that there also exists an indirect effect of area deprivation on the prevalence of hypertension. It can therefore be expected that persons ageing in deprived areas will be at greater risk of hypertension, irrespective of their individual characteristics. The future planning and allocation of primary healthcare in northeastern Germany would therefore greatly benefit from considering the effect of area deprivation.

## Background

Hypertension belongs to one of the most frequent diagnosed chronic conditions in primary healthcare in Germany and within members of the Allgemeine Ortskrankenkasse (AOK health insurance). Current prevalence estimates range between 22% - 51%, depending on the respective study design, study area and definition of hypertension [1–4]. The potential for prevention of severe conditions arising from high blood pressure such as stroke, ischemic heart diseases or kidney failure [5] is high given the strong association to modifiable lifestyle-related factors [2]. Lack of physical exercise [6], sleep deprivation [7], workplace-associated stress [8], smoking [9], excess alcohol consumption [10] and overweight [11] all belong to modifiable risk factors associated with hypertension.

Although lifestyle-related factors on an individual level play a substantial role for the development of chronic conditions, several studies additionally point out that area deprivation is – independent of individual factors – an important determinant for chronic conditions [12–15]. These findings are important for planning of primary healthcare in Germany, which still relies mainly on the ratio of 1671 inhabitants per one general practitioner (GP) at the scale of aggregated municipalities – the so-called - central areas (Mittelbereiche), which was established in the 1990s [16, 17]. However, the discussion about the relevance of area deprivation indices for allocation of healthcare is still relatively young in Germany [18, 19] when compared to other countries such as the United Kingdom [20, 21], Canada [22], France [23] or Spain [24].

In the German health care system, all citizens are required to be enrolled in the statutory health insurance, unless their salary exceeds a certain threshold. In this case, citizens can choose private health insurance or remain voluntarily enrolled in the statutory health insurance [25]. Approximately 86% of the population are covered by statutory health insurance, 10% by private health insurance and 4% are covered by the state [26, 27]. The statutory health insurance provides equal access to healthcare for each insurant, regardless of individual income. Two main principles characterize the German healthcare system: Solidarity and self-government. Solidarity is ensured by a fixed proportion of 15.5% of the personal gross income of each insurant’s salary being deducted as health insurance contribution until a certain income-threshold (Beitragsbemessungsgrenze). Logically, persons with a higher income subsidize persons with a lower income in this system [25]. Self-government refers to the actors of the German healthcare system organizing healthcare in accordance to each other within a legal and political framework set by the government [28].

In the case of primary care, the planning and provision of general practitioners is organized by the association of statutory health insurance physicians in accordance with the respective statutory health insurance providers [29]. As a consequence, it is important for each health insurance provider to analyze the spatial distribution of chronic diseases to be able to engage in evidence-based negotiations with the association of statutory health insurance physicians where new GPs should be allocated. As the revised guidelines of the association of statutory physicians would allow deviations from the ratio of 1671 inhabitants per one GP for areas with high prevalence rates and specific socio-demographic and -economic characteristics [29], it is necessary to detect areas with significantly elevated prevalence rates and to capture the local association between socio-demographic characteristics and the respective chronic disease.

However, analyzing the impact of socio-demographic and -economic characteristics on the prevalence of chronic diseases is challenging based on health insurance claims. Large differences exist in the socio-demographic composition of members of the various health insurance providers with the AOK having the largest proportion of members with a lower socio-economic status and therefore a higher prevalence of chronic diseases [30]. Detailed socio-economic variables such as unemployment rate based on the total population are therefore likely to differ from the unemployment rate within members of a specific health insurance provider. As area deprivation has been shown to have an effect on health independent from individual characteristics [12–15], it is also likely that area deprivation has an effect on the prevalence of chronic conditions, irrespective of the varying socio-demographic composition of members of the respective health insurance providers. As a logical consequence, analyzing the effect of area deprivation could be a more useful approach to capture a location-specific increased medical demand in health insurance claims. However, there are currently no small-scale spatial epidemiological studies published, which evaluate the relevance of the German Index of Multiple Deprivation based on data of a single health insurance provider.

The goal of our study is therefore to (i) analyze the spatial distribution of hypertension based on health insurance claims of northeastern Germany’s largest health insurance provider – the AOK Nordost, (ii) identify possible associations with area deprivation and socio-demographic population characteristics and (iii) analyze how these associations vary over space.

## Methods

### Dependent variable

For this study, we used health insurance claims from the AOK Nordost for 2012, which covers roughly a quarter of the population in the federal states of Berlin, Brandenburg and Mecklenburg-West Pomerania. Of the 1.79 million insurants, 810 thousand (45.2%) were diagnosed with hypertension. We defined hypertension as a confirmed diagnosis with the ICD-codes (10th revision) I10 – I15 [1].

As long as an insurant is treated for hypertension, this diagnosis remains in the insurants personal medical file. The unique insurant number was used to ensure that each insurant is included only once in the analysis.

The data were aggregated to the level of municipalities and urban districts for the cartographic visualization of the sex- and age-standardized rates. Since large urban areas such as Germany’s capital Berlin count only as one municipality, we combined the municipalities with the urban districts of Rostock and the postal codes of Berlin, Potsdam, Brandenburg an der Havel, Schwerin, Frankfurt (Oder) and Cottbus to visualize intra-urban differences. Since the AOK Nordost does not cover the total population, the spatial scale of municipalities for the spatial regression analysis was considered too small. We therefore further aggregated the health insurance claims to the level of association of municipalities, which is the next largest administrative unit after municipalities. The underlying map sources for the municipalities and the association of municipalities were downloaded from the federal agency of cartography and geodesy [31].

### Explanatory variables

**Fig. 1:**
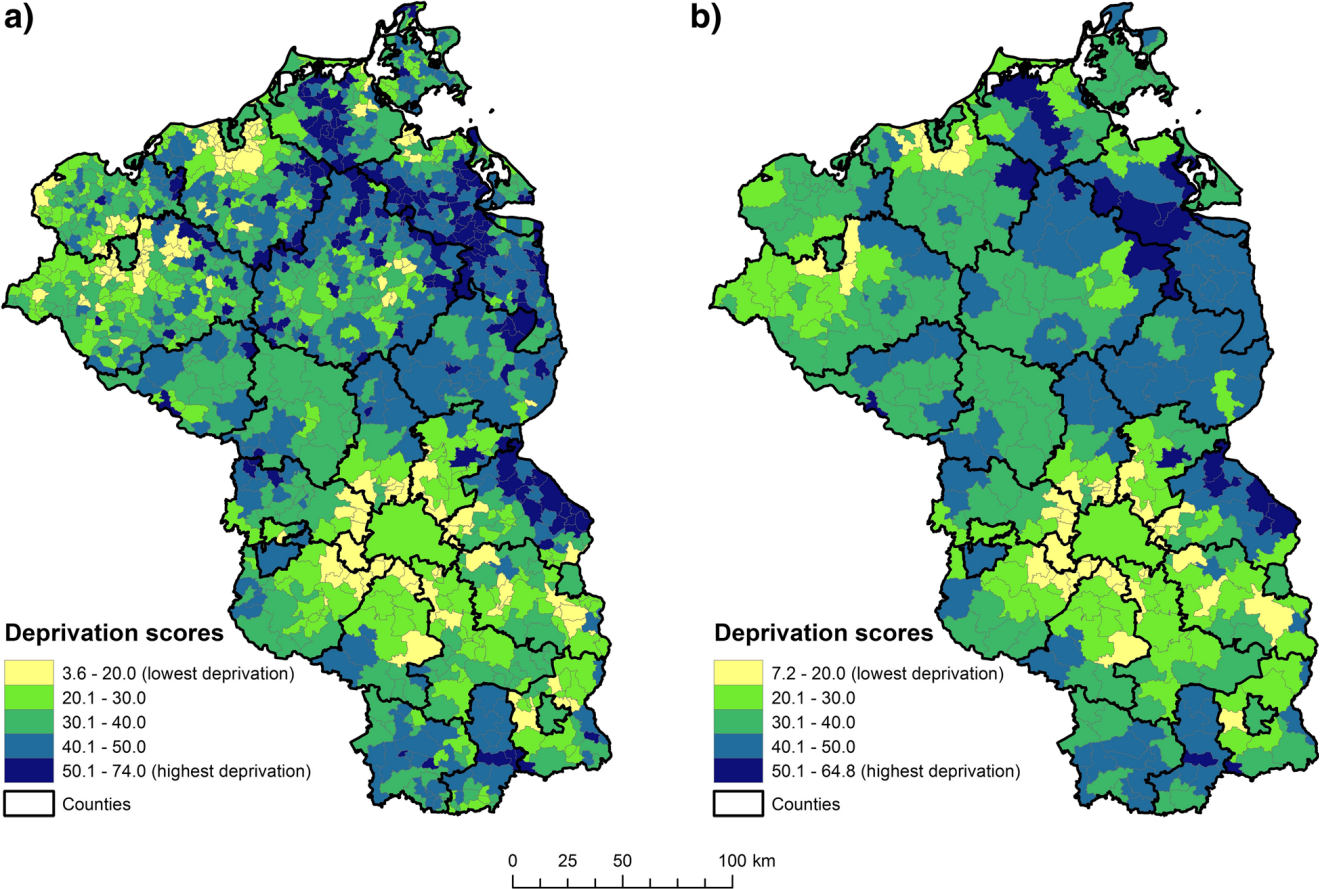
Adaptation of the German Index of Multiple Deprivation at the level of **a** municipalities and **b** association of municipalities for the area covered by the AOK Nordost. Data source: Helmholtz Zentrum München, German Research Center for Environmental Health. Map source: Federal agency of cartography and geodesy

As the aim of our analysis was to evaluate, whether hypertension is associated with area deprivation, we used the “German Index of Multiple Deprivation” (GIMD), which was obtained from the Institute of Health Economics and Health Care Management at the Helmholtz Zentrum München, German Research Center for Environmental Health (Fig. 1). The index includes indicators on demographic, socio-economic and environmental characteristics related to seven different domains of deprivation (i.e. income, employment, education, municipal revenue, social capital, environment, security) [12, 32]. The original GIMD was available for the year 2010 at the municipality level in Germany. To match the spatial scale of association of municipalities for the regression analysis, the index was aggregated proportional to the inhabitants per municipality in 2010.

To account for the increase of hypertension in middle-aged and elderly persons, we used the proportion of insurants aged 45–64 and older than 65 as demographic variables. As several studies found an association between marital status and chronic diseases [33–35], we included the proportion of unmarried, married, divorced and widowed persons as well as data on household composition and proportion of immigrants as possible additional explanatory variables, which were available from the Census 2011 for Germany.

The proportion of persons commuting to work outside their residential municipality for the year 2012 was additionally included as possible explanatory variable as they comprise an important risk group for hypertension. Long commuting times to work – and thus lower levels of physicial activity but higher levels of stress - have been previously been identified as an important modifiable risk factor for a wide range of adverse health outcomes, including hypertension [36–38].This variable was obtained from the federal Federal Agency of Building and Urban Development (BBSR).

### Statistical analysis

#### Cartographic visualization of sex- and age-standardized prevalence rates

We standardized directly for sex and age, using the World Health Organization (WHO) standard population from 1976 [39]. Our goal was to map the standardized prevalence of hypertension at the smallest spatial scale possible. As the number of insurants varies greatly within the 1449 administrative areas in northeastern Germany, the resulting prevalence rates are highly unstable und unreliable. We therefore applied the conditional autoregressive Besag-York-Mollié (BYM) model without covariates to generate more stable and reliable prevalence rates [40]. The BYM model is a Poisson model where the number of observed cases is the dependent variable and the number of expected cases is the offset variable [41]. The conditional autoregressive prior was assigned as the neighborhood structure of the municipalities, defined in this study as municipalities sharing a common edge or border [42]. The model was fitted using Markov-Chain Monte Carlo simulations. The posterior prevalence estimates were calculated based on 1,000,000 simulations of which the first 100,000 were discarded as burn-in period. The BYM model was fitted using the R package “CARBayes” [41]. The results were then imported in ESRI ArcGIS 10.2 for enhanced visualization.

#### Local cluster analysis

As one of our goals was to detect areas with significantly elevated prevalence rates of hypertension, we applied the spatial scan statistic. Local cluster tests are a frequently used tool in public health to prioritize areas for future preventive interventions [43–45]. In this study, we used the spatial scan statistic (SaTScan). The spatial scan statistic is a local cluster test, which identifies the location and the statistical significance of local clusters [46]. In our analysis, we used a purely spatial Poisson model, where the number of hypertension cases are expected to follow an inhomogeneous Poisson distribution [47]. The input data for this model consisted of the sex- and age-adjusted number of hypertension cases, the number of insurants and the centroid coordinates per administrative area. The spatial scan statistic uses a circular scanning window, which is flexibly in size and moves over the centroid coordinates of the study area. The size of the scanning window can incorporate up to 50% of the population at risk. However, this default setting tends to deliver results of no practical use as the scanning window will also include areas of low risk [45, 48]. We therefore restricted the maximum radius of the scanning window to not exceed 10 km. This was done as we defined 10 km as the maximum bearable driving distance in rural areas to GPs [26]. The statistical significance of local clusters was evaluated using 9999 Monte-Carlo replications. We considered only significant clusters with a *p*-value below the 0.001 threshold.

### Spatial regression modelling

#### Ordinary least squares regression modelling

Before creating a geographically weighted regression model, we first identified all significant explanatory variables through exploratory regression. As the ordinary least squares (OLS) regression model is a linear model, it requires a normal distribution of the dependent variable [49]. We therefore visually evaluated the distribution of the untransformed hypertension prevalence and the log-transformed prevalence. The untransformed prevalence was closer to the normal distribution and was therefore chosen as the final dependent variable in our analysis. To identify a set of meaningful explanatory variables, we used a data-mining tool called exploratory regression in ESRI ArcGIS 10.2. This tool is comparable to a stepwise regression. In our study, this tool was used to identify a set of explanatory variables based on following criteria: (i) The explanatory variables are statistically significant (*p* < 0.05) and are free from multicollinearity as measured by the variance inflation factor (VIF < 7.5) [50]. We then chose the most parsimonious model with a set of explanatory variables, which delivered a plausible explanation of hypertension and created an OLS regression model in ESRI ArcGIS 10.2 to determine residual spatial autocorrelation and the presence of heteroscedasticity.

#### Geographically weighted regression modelling

Our goal was not only to estimate the strength of predictors for hypertension, but also to evaluate how the strength of association between the identified explanatory variables and hypertension varies over the study area. In northeastern Germany, the socio-demographic and -economic composition of the population varies widely across the association of municipalities. Based on the results of a previous study on type 2 Diabetes Mellitus in this area [26], we hypothesized that a geographically weighted regression (GWR) model provides a better explanation for hypertension than a global OLS model.

The data input for the GWR model consisted of the same dependent and explanatory variables as in the OLS model and the centroid coordinates of the association of municipalities. We used the R package “GWmodel” for our calculations of a GWR model [51]. In “GWmodel”, a circular kernel is used to calculate for each coordinate a regression equation. Depending on the kernel function used, the coordinate in the center is the regression point and the coordinates towards the edge of the kernel are then weighted with decreasing intensity [52]. Coordinates outside the kernel receive a weight of zero. In “GWmodel”, the form of the kernel can be either Gaussian, bisquare, exponential, tricube or boxcar, where all data points within the kernel receive the same weight. The kernel can be specified as either a fixed radius in km or an adaptive kernel specified as the number of neighbors. The optimization of bandwidth can be based on Akaike’s corrected Information Criterion (AICc) or Cross Validation (CV). The GWR model itself may be a basic GWR regression or may take the form of a robust GWR, which is robust to outliers in the data [51]. In total, 40 different combinations of kernel function, radius, optimization method and GWR type were compared. To evaluate clustering of the residuals, we used the spdep package in R, where the neighbors were defined as areas sharing a common edge or border [53]. To automatically evaluate all possible 40 GWR combinations and clustering of the residuals, we used R version 3.3.1 [54].

### Ethics **statement**

The data and results used in this study were anonymized and do not contain any personal information. The use of anonymized data for research purposes does not require a vote by an ethics committee or an institutional research board.

## Results

### Spatial distribution of hypertension

**Fig. 2:**
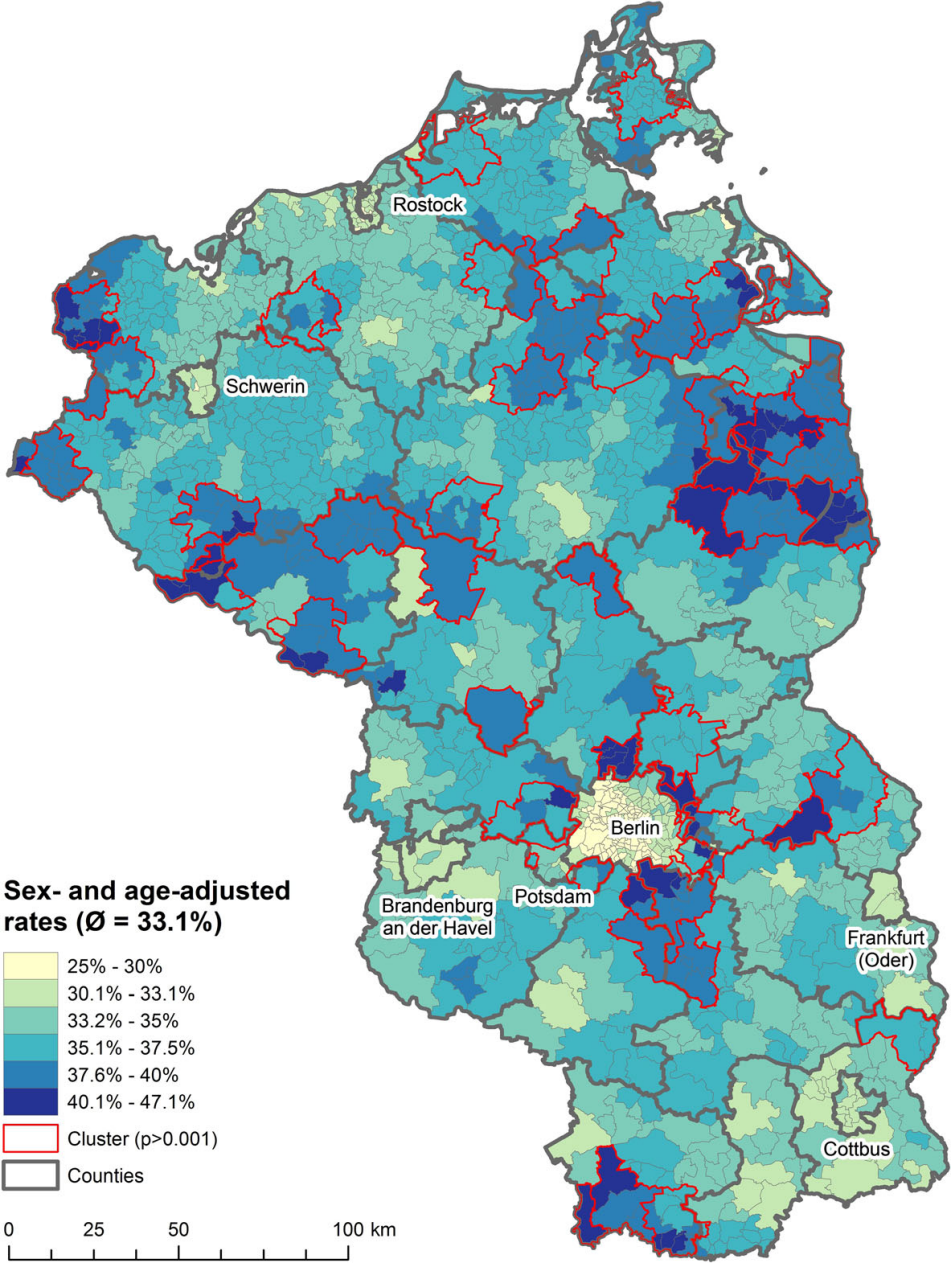
Sex- and age-adjusted prevalence of hypertension in northeastern Germany, 2012. Map source: Federal agency of cartography and geodesy

The overall sex- and age-adjusted hypertension prevalence was 33.1%. The lowest prevalence rates were observed in the urban areas of Berlin, Schwerin, Neubrandenburg and Cottbus (Fig. 2). The highest prevalence rates could be observed in the north of Brandenburg in the counties Uckermark and Prignitz. Local clustering was especially dominant in the commuting belt surrounding Berlin and rural areas of Mecklenburg-West Pomerania.

### Socio-demographic and –economic risk factors of hypertension

We identified four variables as significant predictors of hypertension: (i) Area deprivation, (ii) proportions of insurants aged 45–64, (iii) proportion of insurants aged 65 and older and (iv), proportion of commuters. These four variables explained 67% of the spatial variation of hypertension (Table 1). Multicollinearity was fairly low among the explanatory variables as measured by the variance inflation factor. Since the residuals were significantly clustered, a global OLS model can be considered as inappropriate for modelling the hypertension prevalence.

**Table 1:** Results of the global ordinary least squares regression model

Variable	Coefficient	VIF
intercept	0.0627*	
Insurants aged 45 – 64 (%)	0.0030***	2.2456
Insurants aged 65 and older (%)	0.0083***	2.0878
Area deprivation	0.0005*	1.7968
Commuters (%)	0.0004***	1.5766
Adjusted R^2^	0.67	
AICc	-1309	
Global Moran`s I of residuals	I = 0.168 (*p*<0.001)	

Significance levels: *≤ 0.05; **≤ 0.01: ***≤ 0.001

### Spatially varying risk factors of hypertension

**Fig. 3:**
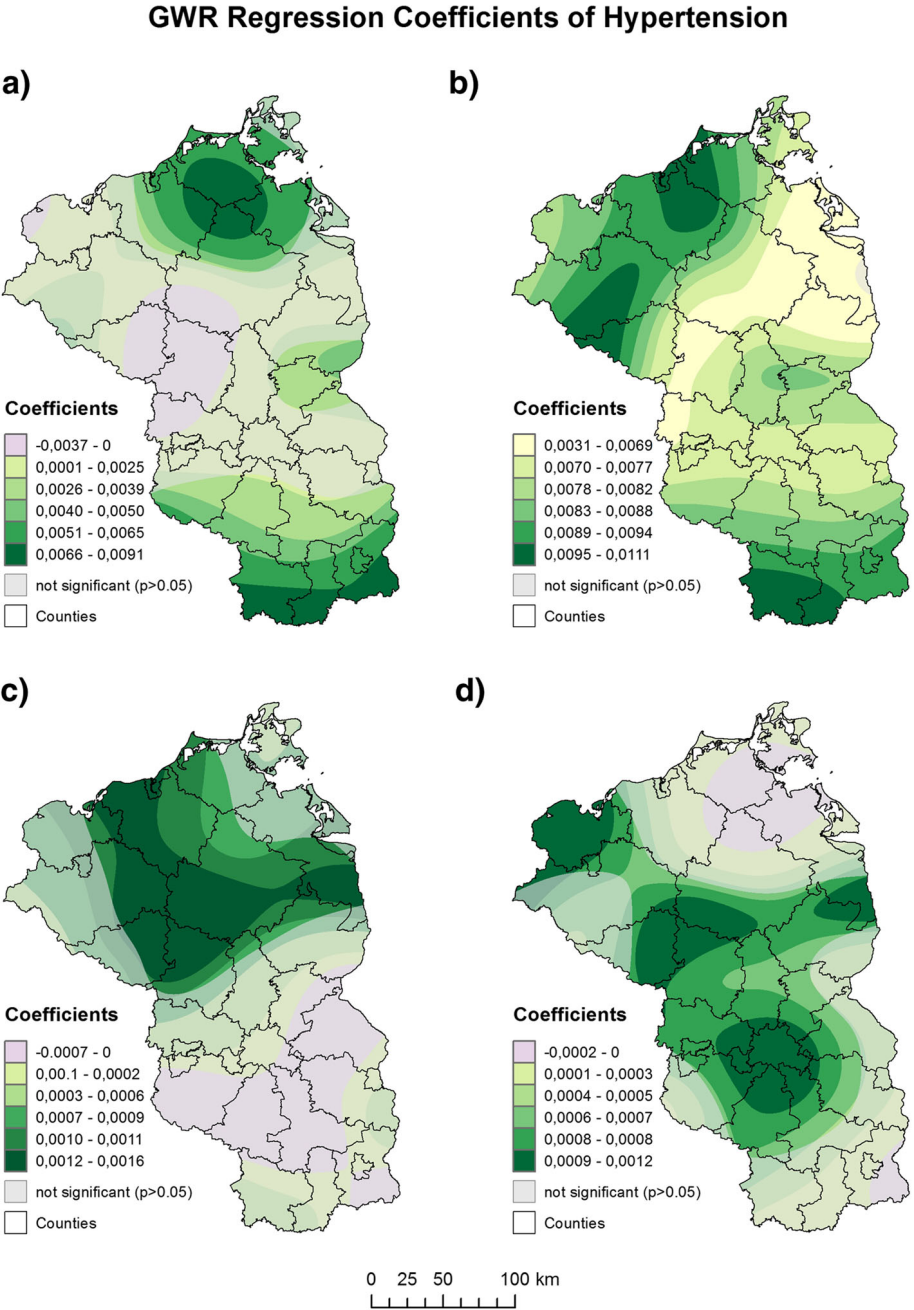
Geographically weighted regression coefficients of hypertension for **a** insurants aged 45–64 years, **b** insurants aged 65 years and older, **c** Area deprivation (GIMD) and **d** commuters. Map source: Federal agency of cartography and geodesy

A fixed Gaussian kernel with an AICc optimized bandwidth of 35 km fulfilled the requirements of the residuals not displaying residual spatial autocorrelation (Moran’s *I* = 0.053; *p* > 0.05). The GWR model outperformed the global OLS model (AICc = −1320) and explained 71% of the spatial variation of hypertension prevalence (adjusted R^2^ = 0.71). The associations between the explanatory variables and hypertension display clear spatial variability (Fig. 3). However, none of the predictor variables was significant in the entire study area.

The proportion of insurants aged 45–64 was only significant in several parts of the study area. The strongest impact was observed in northeastern Mecklenburg-West Pomerania and the southern parts of Brandenburg. A 1 % increase in these will increase the hypertension prevalence by up to 0.91%.

The proportion of insurants aged 65 and older had by far the strongest impact on hypertension. The strongest impact could be observed in the northwestern parts of Mecklenburg-West Pomerania and the southern parts of Brandenburg. One percent increase in insurants aged 65 and older will increase the prevalence by up to 1.1%.

The impact of area deprivation was significant in Mecklenburg-West Pomerania and parts of northern Brandenburg, but not in the larger part of Brandenburg. In these areas, an increase of 1 point of the deprivation score will increase the prevalence by up to 0.16%.

The strongest impact of the proportion of commuters could be observed in proximity to larger urban areas such as Berlin, Brandenburg an der Havel, Wittstock and Wismar. A 1% increase in commuters will increase the prevalence by up to 0.12%.

## Discussion

The prevalence of hypertension varies strongly between the municipalities of northeastern Germany. The risk factors for hypertension consist of proportion of insurants aged 45–64, proportion of insurants aged 65 and older, area deprivation and proportion of persons commuting to work outside their municipality of residence.

The sex- and age-adjusted prevalence of 33.1% is higher than previous estimates based on all statutory health insurants in Germany between 2004 and 2007, which was at 22.3% [1]. This is not surprising, due to the fact that (i) our study area comprises the most deprived areas in whole Germany and that (ii) the percentage of persons with low educational status, low income and therefore a higher prevalence of chronic diseases are higher in our population sample than in the rest of all statutory health insurants [30]. However, the value of a direct comparison with prevalence estimates of other studies is limited due to different ICD-codes used [3] or different study designs [2, 4]. The spatial distribution of hypertension prevalence varied widely between the 1449 municipalities and urban districts in northeastern Germany despite the application of Hierarchical Bayesian smoothing. Local clustering was especially observed in rural areas surrounding Berlin and in parts of Mecklenburg-West Pomerania. Strong regional variations in prevalence estimates for chronic diseases are typical, not only for hypertension [3, 55, 56] but also for a wide range of other diseases such as Diabetes Mellitus [26, 57, 58] or cardiovascular diseases [59, 60]. It is important to note that the spatial distribution of hypertension in northeastern Germany is partially similar to the spatial distribution of type 2 diabetes mellitus [26], which has also been observed in the United States [61] and Germany [3].

In total, we identified the proportion of 45–64 year old insurants, the proportion of insurants older than 65 years, area deprivation and the proportion of persons commuting to work outside of their residential areas as significant predictors for hypertension.

The association of hypertension to the proportion of insurants aged 45 to 64 and 65 and older is not surprising, given the strong increase of hypertension in persons aged 40 and older [62]. However, our results add an important level of detail as the results of GWR point out, that the strength of the association between demographic insurants-characteristics and hypertension varies strongly over space. The proportion of insurants aged 45–64 was not significant in the total study area and the strongest associations could be observed in more deprived areas. Although the proportion of insurants aged 65 and older was significant in almost the entire study area, the strongest associations could also be observed in more deprived areas. The finding that demographic variables display a stronger association to chronic diseases in areas with higher deprivation is supported by previous findings for cardiovascular disease mortality [60] and highlights the use of GWR to analyze location-specific correlations between chronic conditions, demographic variables and area deprivation.

The association between area deprivation and hypertension was only significant in the northern part of Brandenburg and the majority of Mecklenburg-West Pomerania. A significant association between hypertension and lower socio-economic status has been previously noted in many studies [63, 64]. Former studies applying GWR on hypertension also found that the association between socio-economic status and hypertension depends largely on the place of residence [65]. Similar findings were reported for the association between cardiovascular disease and area deprivation [60]. Our results correspond well to these findings, showing that area deprivation is a significant predictor only in a part of the study area.

We found a significant association between the proportions of persons commuting to work outside their residential municipality. Our results clearly show an increase of the strength of this association towards major urban areas such as Berlin, Potsdam and Wismar. Our results are therefore in line with previous studies showing that commuting to work is associated with elevated levels of blood pressure [66–68]. This result is not surprising, given that especially Berlin has a large proportion of persons commuting from Brandenburg to Berlin and from Berlin to surrounding municipalities. In contradiction, all larger cities in Brandenburg have a larger proportion of persons commuting to the city than vice-versa [69], with the majority of commuters travelling between 10 and 30 min to their workplace [70].

### Implications for prevention and provision of healthcare

The results of the BYM disease mapping approach and the cluster analysis employed here point out areas with statistically significant areas with high prevalence rates. These areas could serve as a first and relative exact suggestion where to launch prioritized prevention strategies. The results of GWR additionally point out areas where the proportion of persons commuting to work outside their residential municipality was significant. These findings are of additional use for prevention strategies as commuting to work is one of many potentially modifiable lifestyle-related factors associated with hypertension [36, 71].

As the revised guidelines of the association of statutory physicians would allow deviations from the target ratios of 1671 inhabitants per GPs for areas with peculiar high prevalence rates [29], we have detected such areas in close proximity to Berlin and predominantly rural areas in Mecklenburg-West Pomerania. Given the strong similarity of the spatial distribution of hypertension and type 2 Diabetes Mellitus, it is also likely that other chronic conditions such as stroke and cardiovascular diseases display a similar spatial distribution to that of hypertension and type 2 Diabetes Mellitus [3, 72]. The small-scale variation in prevalence rates suggest that the future allocation of GPs could greatly benefit from smaller spatial scales such as the association of municipalities or even municipalities. This finding is consistent with previous spatial epidemiological research in northeastern Germany [26].

We found that area deprivation has a significant and therefore a direct influence on hypertension only in a fraction of the study area when holding the effect of demographic variables constant. In these areas, the prevalence of hypertension could be directly influenced by deprivation in the form of having a low income, low educational status and fewer job opportunities [63]. On an individual level, a lower socio-economic status is more likely to lead to several lifestyle-related modifiable risk factors such as lack of physical exercise [73] and obesity [74]. However, our results clearly demonstrate that the influence of the proportion of middle-aged and older insurants on the prevalence of hypertension is stronger in more deprived areas – regardless whether area deprivation itself is significantly associated in these areas or not. This corresponds well to previous findings that area deprivation has not only a direct but also an indirect impact on health, irrespective of individual socioeconomic characteristics [12–15]. With the ongoing demographic transition – especially in rural areas of northeastern Germany – it can be expected that persons ageing in deprived areas are more likely to be suffering from chronic diseases than persons ageing in less deprived areas. Results of previous studies have shown that area deprivation has an adverse effect on wellbeing of the aging population [75, 76]. Similarly, other studies applying GWR on cardiovascular diseases mortality found similarly that the effect of proportion of persons aged 65 and older is stronger in more deprived areas [60]. It is important to note that several authors see the use of GWR as rather exploratory due to possible local multicollinearity issues [77] and problems associated with multiple testing [78]. With recent further developments of GWR through the “GWmodel” package – including functions to detect local multicollinearity [51], we see GWR shifting from a rather exploratory approach towards a sophisticated spatial regression modelling approach. Despite still persisting limitations, the application of GWR provides important insights where associations are stronger than elsewhere – an important shortcoming of traditional global spatial regression models, which typically estimate only one single coefficient per explanatory variable [53].

### Strengths and limitations

#### Strengths

First, we used a large database of health insurance claims from northeastern Germany’s largest statutory health insurance provider, consisting of 1.8 million individual insurants in 2012. Thus, our results are representative for a quarter of northeastern Germany’s population.

Second, we displayed the prevalence of hypertension at the smallest possible spatial scale in northeastern Germany. Additionally, we analyzed possible associations between hypertension, area deprivation and socio-demographic population characteristics at the second-smallest spatial scale possible. Our study adds therefore a new level of detail to small-scale spatial epidemiological studies in Germany, which are focused mainly on the spatial scale of counties [17–19, 79, 80].

Third, the use of GWR strongly improved the interpretational capabilities of a spatial regression model as it highlighted how and where the strength of associations between hypertension and the predictor variables varied across the study region.

Fourth, by using area deprivation instead of socio-economic variables based on the insurants of the AOK Nordost, our results can independently be reproduced by different health insurance providers. This in turn could facilitate negotiations about the allocation of healthcare with the association of statutory physicians.

#### Limitations

Our study has several acknowledgeable limitations:

First, our results are representative only for members of the AOK Nordost health insurance and are not necessarily representative for the whole population of northeastern Germany. Large differences exist in the socio-demographic composition of members of the various statutory health insurances, with the AOK in general having the largest proportion of persons with low levels of education and low income [30].

Second, only the diagnosis as ICD-10 code is available in the insurance database. It is unclear whether the treating GP applied the definitions for hypertensions used in the German Health Interview and Examination Survey for Adults (Studie zur Gesundheit Erwachsener in Deutschland; DEGS1) of the leading institution of the German government in the field of biomedicine, the Robert Koch-Institute (RKI) [2] or used a different criterion for hypertension. It is therefore unknown how the prevalence in the health insurance claims database would differ when only the definitions for hypertension by the RKI are applied.

Third, the proportion of persons commuting to work outside their residential municipality is taken from the BBSR and is therefore only available for the whole population of the respective administrative unit. Since the AOK Nordost accounts for approximately a quarter of the population of northeastern Germany, It is currently unknown whether the proportion of commuters within members of the AOK Nordost differs from the proportion among the total population. Given the socio-economic and geographic characteristics of northeastern Germany, it can however be assumed that the proportion of commuters among the AOK Nordost insurants will not differ substantially from the general population.

Fourth, by using the German Index of Multiple Deprivation, we obscured fine socio-economic population characteristics such as employment rate or type of occupation in specific age-groups, which might be potentially of more use to identify the main population at risk for hypertension. For preventive interventions where more detailed socio-economic characteristics are of interest, the analysis should be repeated with health insurance-specific socio-economic variables such as type of insurance, age-specific unemployment rate, occupation and income.

Fifth, the associations examined in this study are based on an ecological study design. In how far these associations are applicable at an individual level as well is subject to further research.

Sixth, the proportion of AOK Nordost insurants is heterogeneously distributed in northeastern Germany with more persons being insured in more deprived areas. It is therefore possible that the effect of area deprivation on hypertension is alleviated in our population sample, given that the proportion of AOK Nordost insurants increases with area deprivation.

Seventh, although the awareness of hypertension is generally very high in Germany with over 80% [2], there is still potential for underdiagnosis, especially in persons with a higher education. In Germany, a routine health check-up (“check-up 35”) is offered every two years to all persons aged 35 and over to detect possible cardiovascular diseases as early as possible. Despite substantial effort of health insurance providers to motivate their insurants to participate in these routine health check-ups, only a third of all eligible persons attends every two years and only approximately 80% of all insurants have ever participated in these routine check-ups. Persons who chose not to participate were on average younger and had a higher education [81]. In how far this may affect the results of our study could not be quantified.

## Conclusions

This study examined the spatial distribution of hypertension based on health insurance claims at the smallest administrative units available. The results of our study clearly show that hypertension varies at the level of municipalities and urban districts in northeastern Germany and highlight the potential of “big data” from health insurance providers for planning and allocation of primary healthcare. The GWR analysis has shown that hypertension is associated with location-specific socio-demographic population characteristics and area deprivation. The future planning of healthcare should therefore be planned at smaller spatial scales than the central areas at which it is currently planned and should reflect the location-specific impact of area deprivation on the prevalence of chronic diseases.

## Abbreviations

AICc: Akaike’s corrected Information Criterion
AOK: Allgemeine Ortskrankenkasse
BBSR: Federal Agency of Building and Urban Development
BYM: Besag–York–Mollié model
CV: Cross validation
DEGS1: Studie zur Gesundheit Erwachsener in Deutschland
GIMD: German Index of Multiple Deprivation
GP: General practitioner
GWR: Geographically weighted regression
ICD: International classification of disease
Km: Kilometer
OLS: Ordinary least squares
RKI: Robert Koch-Institute
VIF: Variance inflation factor
WHO: World Health Organization

## References

[1] Berger I, Horenkamp-Sonntag D, Leipnitz K, Reschke P, Tillmanns H (2009). Bericht zur Schätzung der Morbiditätsveränderung 2008/2009 und zur Repräsentativität und Plausibilität der Datengrundlage des Bewertungsausschusses.

[2] Neuhauser PDH, Thamm M, Ellert U (2013). Blutdruck in Deutschland 2008–2011. Bundesgesundheitsblatt-Gesundheitsforschung-Gesundheitsschutz.

[3] Erhart M, Hering R, Schulz M, von Stillfried DG: Morbiditätsatlas Hamburg. Gutachten zum kleinräumigen Versorgungsbedarf in Hamburg–erstellt durch das Zentralinstitut für die kassenärztliche Versorgung in Deutschland, im Auftrag der Behörde für Gesundheit und Verbraucherschutz Hamburg «Hamburg 2013, 7.

[4] van den Berg N, Meinke-Franze C, Fiss T, Baumeister SE, Hoffmann W (2013). Prevalence and determinants of controlled hypertension in a German population cohort. BMC Public Health.

[5] Lawes CM, Vander Hoorn S, Rodgers A (2008). Global burden of blood-pressure-related disease, 2001. Lancet.

[6] Warburton DE, Nicol CW, Bredin SS (2006). Health benefits of physical activity: the evidence. Can Med Assoc J.

[7] Mullington JM, Haack M, Toth M, Serrador JM, Meier-Ewert HK (2009). Cardiovascular, inflammatory, and metabolic consequences of sleep deprivation. Prog Cardiovasc Dis.

[8] Yang H, Schnall PL, Jauregui M, T-C S, Baker D (2006). Work hours and self-reported hypertension among working people in California. Hypertension.

[9] Talukder MH, Johnson WM, Varadharaj S, Lian J, Kearns PN, El-Mahdy MA, Liu X, Zweier JL: Chronic cigarette smoking causes hypertension, increased oxidative stress, impaired NO bioavailability, endothelial dysfunction, and cardiac remodeling in mice. American Journal of Physiology-Heart and Circulatory Physiology 2010:ajpheart. 00868.02010. 10.1152/ajpheart.00868.2010 PMC3023256 21057039

[10] Klatsky AL (2003). Alcohol and hypertension: does it matter? Yes. J Cardiovasc Risk.

[11] Chiolero A, Cachat F, Burnier M, Paccaud F, Bovet P (2007). Prevalence of hypertension in schoolchildren based on repeated measurements and association with overweight. J Hypertens.

[12] Maier W, Holle R, Hunger M, Peters A, Meisinger C, Greiser K, Kluttig A, Völzke H, Schipf S, Moebus S (2013). The impact of regional deprivation and individual socio-economic status on the prevalence of type 2 diabetes in Germany. A pooled analysis of five population-based studies. Diabet Med.

[13] Grintsova O, Maier W, Mielck A (2014). Inequalities in health care among patients with type 2 diabetes by individual socio-economic status (SES) and regional deprivation: a systematic literature review. Int J Equity Health.

[14] Olives C, Myerson R, Mokdad AH, Murray CJ, Lim SS (2013). Prevalence, awareness, treatment, and control of hypertension in United States counties, 2001–2009. PLoS One.

[15] Skapinakis P, Lewis G, Araya R, Jones K, Williams G (2005). Mental health inequalities in Wales, UK: multi-level investigation of the effect of area deprivation. Br J Psychiatry.

[16] Bedarfsplanungs - Richtlinie Stand: 15. Oktober 2015 des Gemeinsamen Bundesausschusses über die Bedarfsplanung sowie die Maßstäbe zur Feststellung von Überversorgung und Unterversorgung in der vertragsärztlichen Versorgung [https://www.g-ba.de/downloads/62-492-1109/BPL-RL_2015-10-15_iK-2016-01-06.pdf].

[17] Ozegowski S, Sundmacher L (2012). Wie, bedarfsgerecht ‘ist die Bedarfsplanung? Eine Analyse der regionalen Verteilung der vertragsärztlichen Versorgung. Gesundheitswesen.

[18] Hofmeister C, Maier W, Mielck A, Stahl L, Breckenkamp J, Razum O (2016). Regionale Deprivation in Deutschland: Bundesweite Analyse des Zusammenhangs mit Mortalität unter Verwendung des ,German Index of Multiple Deprivation (GIMD). Das Gesundheitswesen.

[19] Kopetsch T, Maier W, Analyse d. Zusammenhangs zwischen regionaler Deprivation und Inanspruchnahme–Ein Diskussionsbeitrag zur Ermittlung des Arztbedarfes in Deutschland. Das Gesundheitswesen. 2016; 10.1055/s-0042-100622 27171729

[20] Morris R, Carstairs V (1991). Which deprivation? A comparison of selected deprivation indexes. Journal of Public Health.

[21] Noble M, Wright G, Smith G, Dibben C (2006). Measuring multiple deprivation at the small-area level. Environ Plan A.

[22] Pampalon R, Raymond G (2000). A deprivation index for health and welfare planning in Quebec. Chronic Diseases and Injuries in Canada.

[23] Havard S, Deguen S, Bodin J, Louis K, Laurent O, Bard D (2008). A small-area index of socioeconomic deprivation to capture health inequalities in France. Soc Sci Med.

[24] Ocaña-Riola R, Saurina C, Fernández-Ajuria A, Lertxundi A, Sánchez-Cantalejo C, Saez M, Ruiz-Ramos M, Barceló M, March JC, Martínez J (2008). Area deprivation and mortality in the provincial capital cities of Andalusia and Catalonia (Spain**)**. J Epidemiol Community Health.

[25] Obermann K, Müller P, Müller H-H, Schmidt B, Glazinski B (2013). The German health care system: a concise overview: Ratgeber-Verlag.

[26] Kauhl B, Schweikart J, Krafft T, Keste A, Moskwyn M (2016). Do the risk factors for type 2 diabetes mellitus vary by location? A spatial analysis of health insurance claims in northeastern Germany using kernel density estimation and geographically weighted regression. Int J Health Geogr.

[27] Ziegler U, Doblhammer G (2009). Prävalenz und Inzidenz von Demenz in Deutschland–Eine Studie auf Basis von Daten der gesetzlichen Krankenversicherungen von 2002. Das Gesundheitswesen.

[28] Bärnighausen T, Sauerborn R (2002). One hundred and eighteen years of the German health insurance system: are there any lessons for middle-and low-income countries?. Soc Sci Med.

[29] Die neue Bedarfsplanung Grundlagen, Instrumente und regionale Möglichkeiten [http://www.kbv.de/media/sp/Instrumente_Bedarfsplanung_Broschuere.pdf].

[30] Schnee M. Sozioökonomische Strukturen und Morbidität in den gesetzlichen Krankenkassen. Gesundheitsmonitor. 2008:88–104.

[31] Verwaltungsgebiete mit Einwohnerzahlen [http://www.geodatenzentrum.de/geodaten/gdz_rahmen.gdz_div?gdz_spr=deu&gdz_akt_zeile=5&gdz_anz_zeile=1&gdz_unt_zeile=15&gdz_user_id=0].

[32] Maier W, Fairburn J, Mielck A (2012). Regional deprivation and mortality in Bavaria. Development of a community-based index of multiple deprivation. Gesundheitswesen (Bundesverband der Arzte des Offentlichen Gesundheitsdienstes (Germany)).

[33] Kaplan RM, Kronick RG (2006). Marital status and longevity in the United States population. J Epidemiol Community Health.

[34] Holt-Lunstad J, Birmingham W, Jones BQ (2008). Is there something unique about marriage? The relative impact of marital status, relationship quality, and network social support on ambulatory blood pressure and mental health. Ann Behav Med.

[35] Manzoli L, Villari P, Pirone GM, Boccia A (2007). Marital status and mortality in the elderly: a systematic review and meta-analysis. Soc Sci Med.

[36] Schaefer A (2005). Commuting takes its toll. Scientific American Mind.

[37] Hu G, Pekkarinen H, Hänninen O, Yu Z, Guo Z, Tian H (2002). Commuting, leisure-time physical activity, and cardiovascular risk factors in China. Med Sci Sports Exerc.

[38] Hansson E, Mattisson K, Björk J, Östergren P-O, Jakobsson K (2011). Relationship between commuting and health outcomes in a cross-sectional population survey in southern Sweden. BMC Public Health.

[39] Ahmad OB, Boschi-Pinto C, Lopez AD, Murray CJ, Lozano R, Inoue M. Age standardization of rates: a new WHO standard. Geneva: World Health Organization. 2001;9

[40] Besag J, York J, Mollié A (1991). Bayesian image restoration, with two applications in spatial statistics. Ann Inst Stat Math.

[41] Lee D, Lee MD (2016). Package ‘CARBayes’.

[42] Roger S, Bivand EP, Gomez-Rubio V (2013). Applied spatial data analysis with R.

[43] Tanser F, Bärnighausen T, Cooke GS, Newell M-L. Localized spatial clustering of HIV infections in a widely disseminated rural South African epidemic. International Journal of Epidemiology. 2009:dyp148. 10.1093/ije/dyp148 PMC2720393 19261659

[44] Coleman M, Coleman M, Mabuza AM, Kok G, Coetzee M, Durrheim DN (2009). Using the SaTScan method to detect local malaria clusters for guiding malaria control programmes. Malar J.

[45] Kauhl B, Heil J, Hoebe CJ, Schweikart J, Krafft T, Dukers-Muijrers NH (2015). The spatial distribution of hepatitis C virus infections and associated determinants—an application of a geographically weighted poisson regression for evidence-based screening interventions in hotspots. PLoS One.

[46] Kulldorff M: SaTScan user guide for version 9.0. Department of Ambulatory Care and Prevention, Harvard Medical School, Boston, M*A* 2010.

[47] Kulldorff M (1997). A spatial scan statistic. Communications in Statistics-Theory and methods.

[48] Chen J, Roth RE, Naito AT, Lengerich EJ, MacEachren AM (2008). Geovisual analytics to enhance spatial scan statistic interpretation: an analysis of US cervical cancer mortality. Int J Health Geogr.

[49] Poole MA, O'Farrell PN. The assumptions of the linear regression model. Trans Inst Br Geogr. 1971:145–58.

[50] How Exploratory Regression works [http://desktop.arcgis.com/en/arcmap/10.3/tools/spatial-statistics-toolbox/how-exploratory-regression-works.htm].

[51] Lu B, Harris P, Charlton M, Brunsdon C (2014). The GWmodel R package: further topics for exploring spatial heterogeneity using geographically weighted models. Geo-spatial Information Science.

[52] Fotheringham AS, Brunsdon C, Charlton M: Geographically weighted regression: John Wiley & Sons, Limited; 2003.

[53] Bivand R, Altman M, Anselin L, Assunção R, Berke O (2016). Package ‘spdep’.

[54] Team RC: R: A language and environment for statistical computing. R Foundation for Statistical Computing, Vienna, Austria. 2013. In*.*: ISBN 3–900051–07-0; 2014.

[55] Wang Z, Du Q, Liang S, Nie K, Lin D-N, Chen Y, Li J-J (2014). Analysis of the spatial variation of hospitalization admissions for hypertension disease in Shenzhen, China. Int J Environ Res Public Health.

[56] Grobe TG: Von Daten mit geografischen Punktzuordnungen zu Kartendarstellungen-ein (fast) universelles Makro.

[57] Dijkstra A, Janssen F, De Bakker M, Bos J, Lub R, Van Wissen LJ, Hak E (2013). Using spatial analysis to predict health care use at the local level: a case study of type 2 diabetes medication use and its association with demograpHic change and socioeconomic status. PLoS One.

[58] Siordia C, Saenz J, Tom SE (2012). An introduction to macro-level spatial nonstationarity: a geographically weighted regression analysis of diabetes and poverty. Human geographies.

[59] Asaria P, Fortunato L, Fecht D, Tzoulaki I, Abellan JJ, Hambly P, de Hoogh K, Ezzati M, Elliott P (2012). Trends and inequalities in cardiovascular disease mortality across 7932 English electoral wards, 1982–2006: Bayesian spatial analysis. Int J Epidemiol.

[60] Ford MM, Highfield LD (2016). Exploring the spatial association between social deprivation and cardiovascular disease mortality at the neighborhood level. PLoS One.

[61] Schlundt DG, Hargreaves MK, McClellan L (2006). Geographic clustering of obesity, diabetes, and hypertension in Nashville, Tennessee. The Journal of ambulatory care management.

[62] Janhsen K, Strube H, Starker A (2008). Themenheft 43" Hypertonie".

[63] Grotto I, Huerta M, Sharabi Y (2008). Hypertension and socioeconomic status. Curr Opin Cardiol.

[64] Keenan NL, Rosendorf KA, Control CfD, Prevention (2011). Prevalence of hypertension and controlled hypertension—United States, 2005–2008. MMWR Surveill Summ.

[65] Park S-Y, Kwak J-M, Seo E-W, Lee K-S. Spatial analysis of the regional variation of hypertensive disease mortality and its socio-economic correlates in South Korea. Geospat Health. 2016;11(2) 10.4081/gh.2016.420 27245801

[66] Lyons G, Chatterjee K (2008). A human perspective on the daily commute: costs, benefits and trade-offs. Transp Rev.

[67] Hoehner CM, Barlow CE, Allen P, Schootman M (2012). Commuting distance, cardiorespiratory fitness, and metabolic risk. Am J Prev Med.

[68] Hilbrecht M, Smale B, Mock SE (2014). Highway to health? Commute time and well-being among Canadian adults. World Leisure Journal.

[69] Bogai D, Seibert H, Wiethölter D. Weiter zunehmende Mobilität als Strategie gegen Erwerbslosigkeit: Institut für Arbeitsmarkt-und Berufsforschung der Bundesagentur für. Arbeit. 2006;

[70] Winkelmann U (2010). Manche pendeln weit “–Berufspendler im Bundesländervergleich”. Statistisches Monatsheft Baden-Württemberg.

[71] Glehr R, Danzinger K (2014). Hypertonie aus der Sicht des niedergelassenen Allgemeinmediziners. Journal für Hypertonie-Austrian J Hypertens.

[72] Barker LE, Kirtland KA, Gregg EW, Geiss LS, Thompson TJ (2011). Geographic distribution of diagnosed diabetes in the US: a diabetes belt. Am J Prev Med.

[73] Schneider S, Becker S (2005). Prevalence of physical activity among the working population and correlation with work-related factors: results from the first German National Health Survey. J Occup Health.

[74] McLaren L (2007). Socioeconomic status and obesity. Epidemiol Rev.

[75] Gilleard C, Hyde M, Higgs P (2007). The impact of age, place, aging in place, and attachment to place on the well-being of the over 50s in England. Research on Aging.

[76] Breeze E, Jones D, Wilkinson P, Bulpitt C, Grundy C, Latif A, Fletcher A (2005). Area deprivation, social class, and quality of life among people aged 75 years and over in Britain. Int J Epidemiol.

[77] Wheeler D, Tiefelsdorf M (2005). Multicollinearity and correlation among local regression coefficients in geographically weighted regression. J Geogr Syst.

[78] Byrne G, Charlton M, Fotheringham S: Multiple dependent hypothesis tests in geographically weighted regression. In: *Proceedings of the 10th International Conference on GeoComputation:* 2009: University of New South Wales; 2009.

[79] Weyermann M, Knorr S, Czihal T, von Stillfried D, Drösler S: Qualitätsindikatoren zu potentiell vermeidbaren Krankenhausaufnahmen bei chronischen Erkrankungen: Determinanten der kleinräumigen Variabilität unter Berücksichtigung der Erkrankungsprävalenz. Zeitschrift für Palliativmedizin 2014, 15(03):PD287.

[80] Pollmanns J, Drösler S, Knorr S, Weyermann M (2014). Kleinräumige Verteilung vermeidbarer Krankenhausaufnahmen bei Hypertonie und Herzinsuffizienz in Deutschland. Zeitschrift für Palliativmedizin.

[81] Sönnichsen A, Sperling T, Donner-Banzhoff N, Baum E (2007). Unterschiede zwischen Teilnehmern und Nichtteilnehmern an der Gesundheitsuntersuchung. ZFA-Zeitschrift für Allgemeinmedizin.

